# *Citrus hassaku* Extract Powder Increases Mitochondrial Content and Oxidative Muscle Fibers by Upregulation of PGC-1α in Skeletal Muscle

**DOI:** 10.3390/nu13020497

**Published:** 2021-02-03

**Authors:** Shiori Akashi, Akihito Morita, Yusuke Mochizuki, Fuka Shibuya, Yasutomi Kamei, Shinji Miura

**Affiliations:** 1 Laboratory of Nutritional Biochemistry, Graduate School of Nutritional and Environmental Sciences, University of Shizuoka, Shizuoka 422-8526, Japan; s19225@u-shizuoka-ken.ac.jp (S.A.); moritaa@u-shizuoka-ken.ac.jp (A.M.); us1626215@gmail.com (Y.M.); tdtapco@outlook.jp (F.S.); 2 Laboratory of Molecular Nutrition, Graduate School of Life and Environmental Sciences, Kyoto Prefectural University, Kyoto 606-8522, Japan; kamei@kpu.ac.jp

**Keywords:** auraptene, 7-geranyloxycoumarin, peroxisome proliferator-activated receptor-γ coactivator-1α, mitochondria, skeletal muscle, muscle fiber, fruit, citrus fruit

## Abstract

Peroxisome proliferator-activated receptor-γ coactivator-1α (PGC-1α) is expressed in skeletal muscles and regulates systemic metabolism. Thus, nutraceuticals targeting skeletal muscle PGC-1α have attracted attention to modulate systemic metabolism. As auraptene contained in citrus fruits promotes lipid metabolism and improves mitochondrial respiration, it could increase mitochondrial function through PGC-1α. Therefore, we hypothesized that PGC-1α is activated by auraptene and investigated its effect using *Citrus hassaku* extract powder (CHEP) containing >80% of auraptene. C2C12 myotubes were incubated with vehicle or CHEP for 24 h; C57BL/6J mice were fed a control diet or a 0.25% (*w*/*w*) CHEP-containing diet for 5 weeks. PGC-1α protein level and mitochondrial content increased following CHEP treatment in cultured myotubes and skeletal muscles. In addition, the number of oxidative fibers increased in CHEP-fed mice. These findings suggest that CHEP-mediated PGC-1α upregulation induced mitochondrial biogenesis and fiber transformation to oxidative fibers. Furthermore, as CHEP increased the expression of the protein sirtuin 3 and of phosphorylated AMP-activated protein kinase (AMPK) and the transcriptional activity of PGC-1α, these molecules might be involved in CHEP-induced effects in skeletal muscles. Collectively, our findings indicate that CHEP mediates PGC-1α expression in skeletal muscles and may serve as a dietary supplement to prevent metabolic disorders.

## 1. Introduction

Peroxisome proliferator-activated receptor-γ coactivator-1α (PGC-1α) functions as an inducible co-regulator of energy homeostasis [1,2,3,4]. It is abundantly expressed in tissues with high energy demand, including skeletal muscle, brown adipose tissue, heart, kidney, and brain [5]. PGC-1α affects energy metabolism by interacting with transcription factors such as peroxisome proliferator-activated receptors (PPARs) [6]. Previously, we revealed that overexpression of skeletal muscle-specific PGC-1α increases mitochondrial content, the transformation of glycolytic fibers to oxidative fibers, fatty acid oxidation, and exercise capacity [7] and inhibits the progression of atherosclerosis [8]. Furthermore, a PGC-1α activator which selectively increases PGC-1α expression in myotubes and skeletal muscle increases fatty acid oxidation and ameliorates pyruvate tolerance, glucose tolerance, insulin sensitivity, hyperglycemia, and dyslipidemia [9]. Conversely, dysregulation of PGC-1α is involved in the onset of metabolic diseases, including obesity, diabetes, and cardiovascular diseases [3]. Therefore, upregulation of PGC-1α to modulate metabolic pathways is an interesting strategy for preventing metabolic disorders.

In recent years, nutraceuticals have attracted attention as an alternative medication for disease prevention [10]. For example, resveratrol, a polyphenol, can increase mitochondrial biosynthesis and resistance to muscle fatigue through PGC-1α expression [11]. Furthermore, resveratrol treatment protects mice against diet-induced obesity and insulin resistance [11]. In this study, we focused on *Citrus hassaku* extract powder (CHEP), which is known to contain auraptene (7-geranyloxycoumarin). Auraptene is a prenyloxy coumarin found in the peel of several citrus fruits and, in particular, in peels of *C. hassaku* [12]. Auraptene has numerous pharmacological properties, including anti-cancer, anti-inflammatory, neuroprotective, gastrointestinal-protective, and immune-protective activity [13]. Furthermore, several studies have shown that auraptene improves lipid metabolism disorders [14,15]. Auraptene increases mRNA expression levels of PPARα target genes related to fatty acid oxidation in skeletal muscle and liver, reducing adipocyte size and triglyceride accumulation [14]. As the stimulation of lipid metabolism is involved in the amount and activity of mitochondria [16], it is speculated that auraptene can activate both PPARα and PGC-1α. Moreover, auraptene reportedly affects mitochondrial respiration in dopaminergic neurons by stimulating the expression of transcription factor nuclear factor erythroid 2-like 2 and inducing the expression of downstream genes encoding antioxidant enzymes, including glutathione peroxidase 1 [17]. Furthermore, PGC-1α is required for the induction of several reactive oxygen species (ROS)-detoxifying enzymes, including glutathione peroxidase 1 [18]; hence, PGC-1α is possibly activated by auraptene.

To verify this speculation, we investigated the effect of auraptene on PGC-1α regulation in skeletal muscle using CHEP containing a high concentration of auraptene (80%). In this study, we observed that CHEP increased the expression and activity of PGC-1α in skeletal muscle. Furthermore, we demonstrated that CHEP increased mitochondrial content and the number of oxidative fibers in murine skeletal muscle. Sirtuin 3 (SIRT3), a sirtuin member of the NAD^+^-dependent deacetylase family, was also upregulated by CHEP and may be involved in the mechanism of CHEP-induced effects.

## 2. Materials and Methods

### 2.1. Preparation of CHEP

CHEP, prepared according to a previously described method with slight modifications [12], was kindly provided by ARKRAY (Kyoto, Japan). In brief, peel oil prepared from *C. hassaku* was stored at −20 °C to obtain a precipitate containing auraptene. The crystal fraction was prepared from the precipitate by recrystallization in ethanol. The crystal fraction was recovered by filtration and washed with distilled water. The washed crystals were dried under reduced pressure. A pulverized crystal fraction was used as CHEP.

The auraptene content was determined by high-performance liquid chromatography using a COSMOSIL 5C_18_-AR-II column (4.6 × 250 mm; Nacalai Tesque, Kyoto, Japan). The mobile phase was methanol/water (95:5, *v*/*v*) at a flow rate of 0.3 mL/min. The detection wavelength was 325 nm. The concentration of auraptene, calculated using a standard curve for auraptene, was 82.2% (*w*/*w*).

### 2.2. Cell Culture

Mouse C2C12 myoblast and human embryonic kidney 293T (HEK293T) cell lines were purchased from American Type Culture Collection (Manassas, VA, USA). C2C12 myoblasts and HEK293T cells were cultured in Dulbecco’s modified Eagle’s medium (DMEM; FUJIFILM Wako Pure Chemical Corporation, Osaka, Japan) with 10% fetal bovine serum (FBS; Sigma-Aldrich, Tokyo, Japan) and an antibiotic/antimycotic mixed solution (100 U/mL penicillin G, 100 µg/mL streptomycin, and 0.25 µg/mL amphotericin B) (Nacalai Tesque). Cells were cultured in a humidified atmosphere at 5% CO_2_ and 37 °C; the medium was replaced every two days.

### 2.3. Treatment of C2C12 Cells with CHEP

C2C12 cells were plated at a density of 5.0 × 10^4^ cells/well in a 24-well plate (AS ONE, Osaka, Japan) containing DMEM supplemented with 10% FBS. Upon reaching near 100% confluency, the medium was switched to differentiation medium, i.e., DMEM with 2% horse serum (Thermo Fisher Scientific, Waltham, MA, USA) and the antibiotic/antimycotic solution (Nacalai Tesque). During differentiation, the medium was replaced every two days. Myotube formation was observed normally after approximately 5 days of differentiation. At this time, C2C12 myotubes were incubated with vehicle or with 37 μg/mL CHEP for 24 h. CHEP was added to the cells at a non-toxic concentration by measuring toxicity using the Cytotoxicity LDH Assay Kit-WST (Dojindo Laboratories, Kumamoto, Japan). DMSO (FUJIFILM Wako Pure Chemical Corporation) was used to dissolve the CHEP. The final concentration of DMSO in the medium was 0.1%.

### 2.4. Animals and Diets

Six-week-old male C57BL/6J mice were purchased from Japan SLC (Shizuoka, Japan). Mice were provided free access to water and a normal chow diet (MF; CREA Japan, Tokyo, Japan) and were maintained under a 12 h light/dark cycle at 22 °C.

Control mice (7-week-old) were fed a high-carbohydrate (HC) diet containing 68%, 10%, and 22% of calories from carbohydrate, fat (high oleic safflower oil), and protein, respectively. Mice in the experimental group were fed ad libitum an HC diet containing 0.25% (*w*/*w*) CHEP. The concentration of CHEP was determined according to previous studies [14], assuming that CHEP contained 80% auraptene. After 5 weeks, tissues were dissected, frozen using liquid nitrogen, and stored at −80 °C until analysis. The tissue blocks of the tibialis anterior (TA) were prepared by rapid freezing using isopentane cooled with liquid nitrogen. All animal experiments were approved by the Institutional Animal Care and Use Committee of the University of Shizuoka (No. 195229; date of approval: 29 March 2019).

### 2.5. Western Blot Analysis

Frozen gastrocnemius and C2C12 myotubes were homogenized in RIPA buffer (Merck Millipore, Temecula, CA, USA) containing protease and phosphatase inhibitor cocktails (Nacalai Tesque). The amount of protein in the homogenate was measured using a bicinchoninic acid protein assay kit (Takara Bio, Shiga, Japan). Twenty micrograms of protein were separated on 10% or 12% SDS-PAGE and transferred to nitrocellulose membranes (Bio-Rad Laboratories, Hercules, CA, USA). Membranes were blocked in 5% bovine serum albumin or 5% skim milk powder in Tris-buffered saline with 0.1% detergent (Nacalai Tesque) for 1 h at room temperature (15–25 °C). Thereafter, the membranes were incubated overnight at 4 °C with primary antibodies, followed by incubation with horseradish peroxidase (HRP)-linked secondary antibodies for 1 h at room temperature (15–25 °C). Signals were detected using ECL Prime Western Blotting Detection Reagent (Cytiva, Tokyo, Japan), and their intensities were quantified using a C-DiGit^®^ blot scanner (LI-COR Biosciences, Lincoln, NE, USA).

The following antibodies were used: anti-PGC-1α (sc-517380; Santa Cruz Biotechnology, TX, USA), anti-cytochrome c oxidase subunit IV (COX4) (4850; Cell Signaling Technology, Danvers, MA, USA), anti-SirT3 (5490; Cell Signaling Technology), anti-phospho-AMP-activated protein kinase (AMPK) α (Thr172) (2535; Cell Signaling Technology), and anti-AMPKα (5831; Cell Signaling Technology). Either of the following HRP-linked secondary antibodies were used: anti-mouse immunoglobulin (Ig) G (7076; Cell Signaling Technology) or anti-rabbit IgG (7074P2; Cell Signaling Technology).

### 2.6. Measurement of Citrate Synthase (CS) Activity and Mitochondrial DNA Content

CS activity was measured as previously described [19] and corrected by variating the protein concentration in the homogenate.

To measure mitochondrial DNA content, estimated as the mitochondrial DNA copy number, the mitochondrial gene cytochrome c oxidase subunit II (*COX2*) was normalized by the copy number of the *36B4* gene contained in the nuclear genome, as described previously [20]. The sequences of the PCR primer pairs were as follows: *COX2*, 5′-CCGACTAAATCAAGCAACAGTAACA-3′ and 5′-AAATTTCAGAGCATTGGCCATAG-3′; *36B4*, 5′-GGCCCTGCACTCTCGCTTTC-3′ and 5′-TGCCAGGACGCGCTTGT-3′.

### 2.7. Immunohistochemistry

The tissue blocks from TA were sectioned transversely (10 μm) using a cryostat (Leica Biosystems, Nussloch, Germany) at −25 °C. Cryosections were attached to an adhesive glass slide (Matsunami Glass, Osaka, Japan) and fixed with ice-cold acetone for 5 min. Then, the sections were sequentially incubated with Mouse-on-Mouse Blocking Reagent (Vector Laboratories, Burlingame, CA, USA) and 5% normal goat serum (Thermo Fisher Scientific), followed by overnight incubation with primary antibodies at 4 °C. After incubation, the cryosections were incubated with secondary antibodies for 1 h at room temperature (15–25  °C) and mounted with ProLong^®^ Gold Antifade Mountant (Thermo Fisher Scientific). Image acquisition was performed using a DMi8 inverted microscope (Leica Microsystems, Wetzlar, Germany).

The following antibodies were used: anti-myosin heavy chain (MHC) type I (BA-F8), anti-MHC type IIa (SC-71), anti-MHC type IIb (BF-F3) (Developmental Studies Hybridoma Bank, Iowa, IA, USA), and anti-laminin (L9393; Sigma-Aldrich). Goat anti-mouse IgG2b Alexa 350 (A21140), goat anti-mouse IgG1 Alexa 555 (A21127), goat anti-mouse IgM Alexa 488 (A21042), and goat anti-rabbit IgG (H+L) Alexa 647 (A21244) (Thermo Fisher Scientific) were used as secondary antibodies.

For each fiber type, their total number was counted to calculate the numerical fiber-type composition (type I, blue; type IIa, red; type IIx, black; and type IIb, green). All analyses were performed by a single observer who was blinded to the administered treatment.

### 2.8. Plasmids

For the firefly luciferase reporter assay system, we used the pGL4.35 plasmid (Promega, Madison, WI, USA) containing nine copies of the GAL4 DNA-binding sequence (upstream activating sequence, UAS). In this system, the pNL1.1.PGK plasmid (Promega) was used to determine transfection efficiency. We used pM-PGC-1α, which expresses the fused protein with the Gal4 DNA-binding domain and PGC-1α proteins, as described previously [21].

### 2.9. Reporter Gene Assay

HEK293T cells were plated at a density of 3.4 × 10^4^ cells/well in 96-well half-size white plates (Corning, New York, NY, USA) containing DMEM supplemented with 10% FBS. In this assay, pGL4.35 (25 ng/well), pM-PGC-1α (25 ng/well), and pNL1.1.PGK (0.02 ng/well) plasmids were co-transfected into HEK293T cells using Lipofectamine^®^ 3000 (Thermo Fisher Scientific). After 24 h, the cells were treated with vehicle (DMSO) or CHEP. The final concentration of DMSO in the medium was 0.1%. After more than 24 h, luciferase activity was measured using the Nano-Glo^®^ Dual-Luciferase^®^ Reporter Assay System (Promega).

### 2.10. Statistical Analysis

All data are expressed as the mean ± standard error of the mean (SEM). Statistical analysis was performed using GraphPad Prism version 6.04 (GraphPad Software, La Jolla, CA, USA). One-way analysis of variance (ANOVA) followed by Dunnett’s test was performed to compare three or more groups. Comparisons between two groups were performed using Student’s t-test (equal variance) or Welch’s t-test (unequal variance) following the F-test; *p* < 0.05 was considered significant.

## 3. Results

### 3.1. CHEP Increased the Amount of PGC-1α Protein and Mitochondria in C2C12 Myotubes

**Figure 1:**
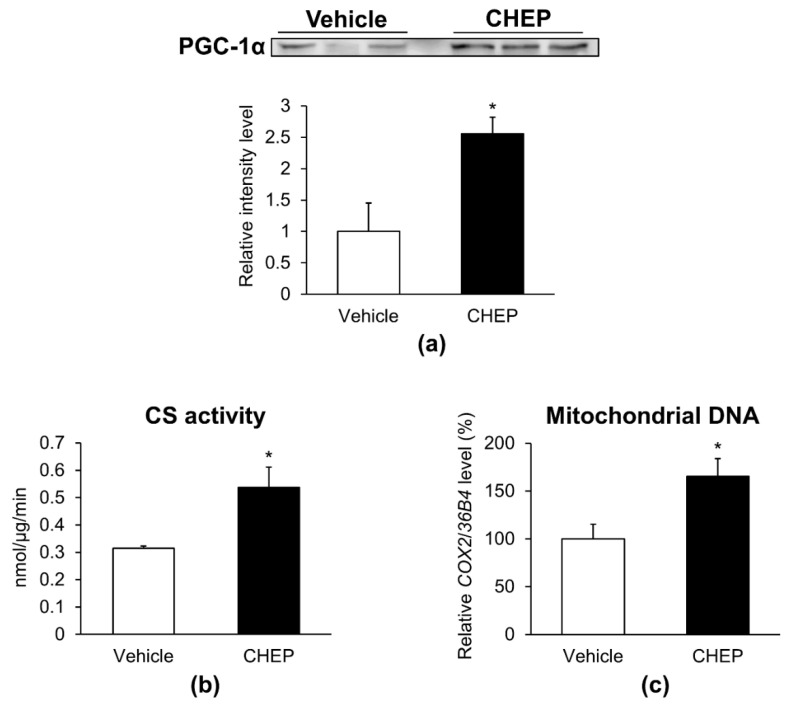
CHEP increases the amount of PGC-1α protein and mitochondria in C2C12 myotubes. Cells were incubated with vehicle (DMSO) or 37 μg/mL of CHEP for 24 h. (**a**) PGC-1α protein expression was evaluated using western blotting. Total PGC-1α protein levels were normalized to the total protein amount in the membrane as obtained after Ponceau S staining. Relative protein levels are expressed compared to those of cells treated with the vehicle. (**b**) CS activity was normalized to the protein content in the homogenate. (**c**) Mitochondrial DNA content, expressed as a percentage with respect to that in vehicle-treated cells. Relative mitochondrial DNA copy number was calculated as the ratio of *COX2* (mitochondrial) to *36B4* (nuclear) gene expression level using real-time PCR. Values are expressed as mean ± SEM (*n* = 3–7); * *p* < 0.05 vs. vehicle. CHEP, *Citrus hassaku* extract powder; PGC-1α, peroxisome proliferator-activated receptor-γ coactivator-1α; CS, citrate synthase; *COX2*, cytochrome c oxidase subunit II; SEM, standard error of the mean.

To determine whether CHEP could increase the amount of PGC-1α in C2C12 myotubes, C2C12 myotubes were incubated with vehicle (DMSO) or CHEP on day 5 of differentiation for 24 h. The amount of total PGC-1α protein, normalized to the total protein amount on the membrane as obtained after Ponceau S staining (Figure S1), was increased by 2.6-fold in cells treated with CHEP compared to those treated with vehicle control (Figure 1a). As an increase in PGC-1α stimulates mitochondrial biogenesis [22], we measured CS activity and mitochondrial DNA content in these cells: both were 1.7-fold higher in cells treated with CHEP than in vehicle-treated cells (Figure 1b,c). These data suggested that CHEP enhanced PGC-1α protein expression and mitochondrial biogenesis in C2C12 myotubes.

### 3.2. CHEP Increased the Amount of PGC-1α Protein, Mitochondria, and Oxidative Muscle Fibers in Murine Skeletal Muscle

**Figure 2:**
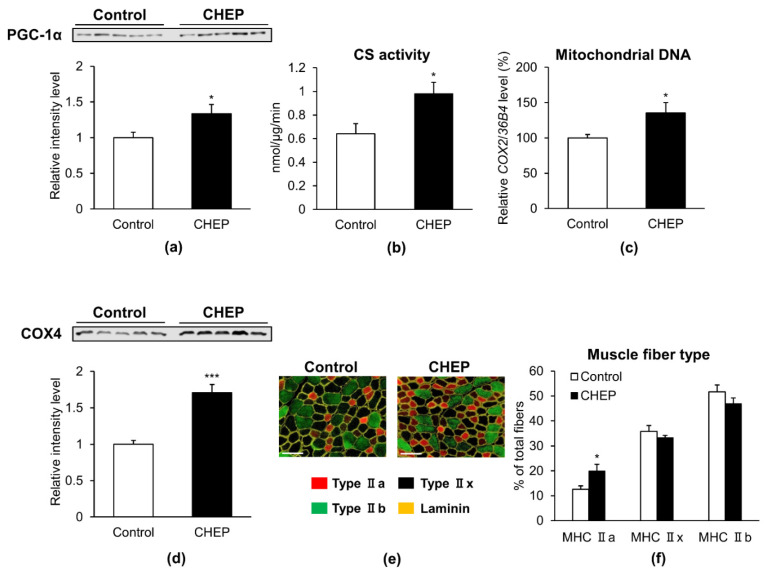
CHEP increases the amount of PGC-1α protein, mitochondria, and oxidative fibers in murine skeletal muscle. Seven-week-old male C57BL/6J mice were treated with an HC diet (control) or an HC diet containing 0.25% (*w*/*w*) CHEP for 5 weeks. Protein expression levels of PGC-1α (**a**) and COX4 (**d**) in the gastrocnemius were evaluated using western blotting. Total PGC-1α and COX4 protein levels were normalized to the total protein amount on the membrane, obtained after Ponceau S staining. Relative protein levels were expressed compared to those of the control. (**b**) CS activity in the gastrocnemius was normalized to protein content in the homogenate. (**c**) Mitochondrial DNA content in the gastrocnemius is expressed as a percentage relative that in control. Relative mitochondrial DNA copy number was calculated as the ratio of *COX2* (mitochondrial) to *36B4* (nuclear) gene expression level, using real-time PCR. (**e**) Representative images of TA stained with anti-MHC type IIa (red), type IIb (green), and laminin (yellow) antibodies. Unlabeled fibers (black) are MHC IIx fibers. There are no MHC I (blue) fibers in this section. Scale bar: 100 μm. (**f**) Percentage of muscle fiber types in TA. Muscle fibers were counted in all cross sections based on immunofluorescence staining. Values are expressed as mean ± SEM (*n* = 5); * *p* < 0.05, *** *p* < 0.001 vs. control. HC, high carbohydrate; COX4, cytochrome c oxidase subunit IV; TA, tibialis anterior; MHC, myosin heavy chain.

To determine whether the effects of CHEP observed in C2C12 myotubes could also be observed in murine skeletal muscle, seven-week-old male C57BL/6J mice were fed an HC diet (control) or an HC diet containing 0.25% (*w*/*w*) CHEP, ad libitum for 5 weeks. Body weights, tissue weights and food intake of these mice were shown in Table S1. As CHEP increased the amount of PGC-1α protein in C2C12 myotubes, PGC-1α protein level increased 1.3-fold in skeletal muscle tissues isolated from mice fed an HC diet containing CHEP (Figure 2a). Furthermore, CS activity, mitochondrial DNA content, and the amount of COX4 protein (another mitochondrial marker protein) increased 1.5-, 1.4-, and 1.7-fold, respectively, in the muscle tissues of mice fed a CHEP-HC diet (Figure 2b–d). These results are consistent with those obtained in C2C12 myotubes, where enhanced PGC-1α expression and mitochondrial content were observed.

As overexpression of PGC-1α in skeletal muscle stimulates the switch of muscle fiber type from glycolytic fibers to oxidative fibers [7], we investigated whether CHEP alters the muscle fiber type in murine skeletal muscle. The composition of each fiber type in muscle tissues was analyzed using MHC type-specific immunohistochemical staining. Figure 2e shows representative immunostaining images for MHC types in muscle from control or CHEP-treated mice. Based on immunohistochemical staining and subsequent counting of all fibers in cross-sections, the number of type IIa fibers was found to be significantly increased in cross sections from CHEP-treated mice compared to that in the sections from control mice (Figure 2f). These results suggest that CHEP induces glycolytic fibers transformation into oxidative-type fibers.

### 3.3. CHEP Promoted SIRT3 Expression and Phosphorylation of AMPK

**Figure 3:**
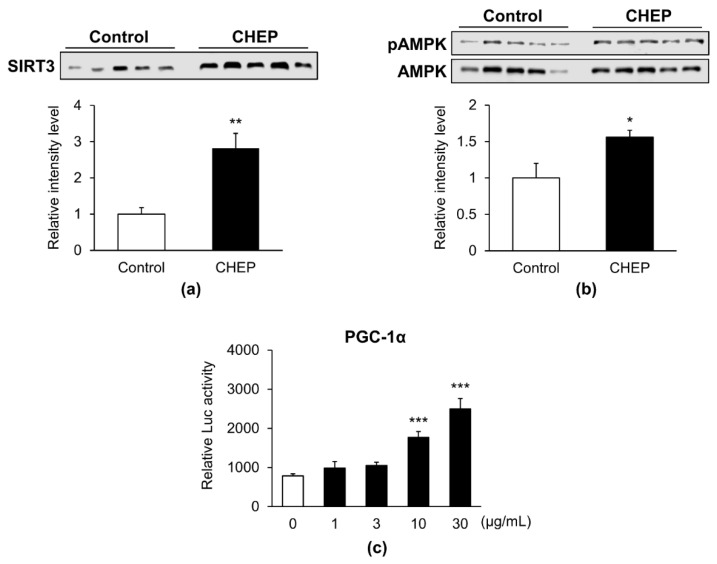
CHEP promotes SIRT3 expression, phosphorylation of AMPK, and PGC-1α transcription activity. Seven-week-old male C57BL/6J mice were fed an HC diet (control) or an HC diet containing 0.25% (*w*/*w*) CHEP, for 5 weeks. (**a**) Total SIRT3 protein levels in the gastrocnemius were measured using western blotting. The levels were normalized to the total protein amount on the membrane, obtained after Ponceau S staining. (**b**) The amount of phospho-AMPK (pAMPK) protein in the gastrocnemius was measured using western blotting. The levels were corrected by the amount of total AMPK protein. Relative protein levels were expressed compared to those of the control. Values are expressed as the mean ± SEM (*n* = 5); * *p* < 0.05, ** *p* < 0.01 vs. control. (**c**) Transcriptional activation of PGC-1α by CHEP was measured using a reporter gene assay. HEK293T cells were co-transfected with pM-PGC-1α, pGL4.35, and pNL1.1.PGK. CHEP, at different concentrations (1, 3, 10, and 30 µg/mL), was added to the culture medium. The transcriptional activity of PGC-1α was analyzed using the Nano-Glo^®^ luciferase assay. Mean values are indicated as the relative luciferase (Luc) activity, calculated as the ratio of firefly luciferase luminescence to NanoLuc luciferase luminescence. Values are expressed as mean ± SEM (*n* = 3); *** *p* < 0.001 vs. 0 µg/mL. SIRT3, sirtuin 3; AMPK, AMP-activated protein kinase; HEK293T, human embryonic kidney 293T.

Reportedly, the expression of PGC-1α is increased by SIRT3 and phosphorylated AMPK [23,24]. Therefore, we measured the protein levels of SIRT3 and phospho-AMPK in skeletal muscles. Seven-week-old male C57BL/6J mice were fed an HC diet (control) or an HC diet containing 0.25% (*w*/*w*) CHEP for 5 weeks, as previously described. The expression levels of SIRT3 protein in CHEP-treated mice increased 2.8-fold compared to those in control mice (Figure 3a). The ratio of phospho-AMPK protein levels to total AMPK protein levels also increased 1.6-fold in CHEP-treated mice (Figure 3b). Furthermore, as AMPK-mediated phosphorylation of PGC-1α activates its transcriptional activity [25], we performed a reporter gene assay to determine whether CHEP promoted the transcriptional activity of PGC-1α. As PGC-1α is a transcriptional coactivator that does not bind directly to DNA, we created a fusion construct of PGC-1α cDNA and Gal4 DNA-binding domain, transfected it into HEK293T cells, and examined whether CHEP enhanced the transcription activity of Gal4 DNA-binding domain-fused PGC-1α protein by (UAS)_9_-luciferase reporter gene assay [21]. Transfected HEK293T cells were incubated with the vehicle (DMSO) or CHEP for 24 h. Accordingly, CHEP dose-dependently increased the transcriptional activity of PGC-1α (Figure 3c). These observations suggested that CHEP promoted SIRT3 expression and AMPK phosphorylation, as well as that these changes were involved in the upregulation of PGC-1α and subsequent effects in murine skeletal muscle. Additionally, CHEP activated the transcriptional activity of PGC-1α, which might enhance SIRT3 expression.

## 4. Discussion

In the present study, we investigated the PGC-1α-mediated effects of CHEP in skeletal muscle, including mitochondrial content and fiber-type switching. Herein, CHEP treatment increased mitochondrial content in cultured myotubes and murine skeletal muscles, suggesting that CHEP stimulated mitochondrial biogenesis. After auraptene (100 mg/kg, p.o.) administration to rats, the maximum plasma concentration was previously reported to be 1.72 ± 0.38 µg/mL [26]. As the content of auraptene in CHEP was 80%, its maximum plasma concentration after administration of 300 mg/kg/day p.o., was supposed to be 5.16 µg/mL in our in vivo study. Instead, the concentration of auraptene in our in vitro study was supposed to be 30 μg/mL, equivalent to six-fold the assumed maximum plasma concentration in our in vivo study.

In skeletal muscles, an increase in the number of oxidative fibers was also observed in mice fed a CHEP-containing diet, suggesting that CHEP is involved in fiber-type switching from glycolytic to oxidative fibers. Furthermore, increased protein levels of PGC-1α were observed following CHEP treatment. As PGC-1α has been shown to elevate mitochondrial content and oxidative muscle fibers in skeletal muscle [7], the CHEP-mediated increase in PGC-1α might be involved in the effects observed in cultured myotubes and murine skeletal muscles in this study. It has been reported that the expression of PGC-1α in skeletal muscles prevents metabolic syndromes such as obesity and diabetes [9], as well as promotes myokine secretion from skeletal muscles and improves systemic energy metabolism [4]. Our findings indicate that CHEP mediates PGC-1α expression in skeletal muscles and may serve as a dietary supplement to prevent metabolic disorders.

PGC-1α activates several transcription factors, including nuclear respiratory factors (NRF-1 and -2), mitochondrial transcription factor A (TFAM), and estrogen-related receptors (ERRs), and induces the transcription of genes associated with mitochondrial biogenesis [27]. CHEP-induced PGC-1α expression and subsequent activation of these transcription factors seemed to be involved in the increased mitochondrial content in cultured myotubes, as well as in murine skeletal muscle.

Skeletal muscle fibers are classified into two types: slow-twitch (type I) and fast-twitch (type IIa, IIx, and IIb) muscle fibers [28]. Slow muscle fibers are rich in mitochondria and their primary source of energy is oxidative metabolism. Fast muscle fibers, such as IIb fibers, are primarily dependent on glycolytic metabolism [28]. Among the fast-twitch muscle fibers, type IIa fibers partly resemble slow-twitch muscle fibers, as they mainly utilize oxidative metabolism [28]. The amount of mitochondria in type IIa fibers is considerably higher than that in type IIx and IIb fibers [28]. Previous studies have reported that PGC-1α promotes fiber-type switching to oxidative fibers by co-activating myocyte enhancer factor 2 (MEF2) C and MEF2D [29]. Indeed, muscle-specific PGC-1α transgenic mice have demonstrated increased oxidative fibers, as well as mitochondria [7]. Accordingly, CHEP may promote fiber transformation from glycolytic to oxidative, in addition to mitochondrial biogenesis, by increasing PGC-1α protein expression in skeletal muscle tissues.

Several studies have revealed that the expression of PGC-1α is upregulated by SIRT3 [23,24,30]. Furthermore, SIRT3 and PGC-1α reportedly stimulate each other’s expression through an ERRα-mediated positive-feedback loop [31]. In the present study, we observed that CHEP increased the expression of SIRT3 and PGC-1α in murine skeletal muscles, suggesting that CHEP-induced expression of PGC-1α synergistically increased the expression of SIRT3. Similar to PGC-1α, SIRT3 expression is induced by exercise and is rich in metabolically active tissues [23]. It has been reported that the promotion of mitochondrial biogenesis and fiber-type switching can be induced by SIRT3. For instance, mitochondrial biogenesis is enhanced by SIRT3 overexpression in C2C12 myotubes [31], and mitochondrial density and formation of oxidative muscle fibers increase in skeletal muscle-specific-SIRT3-overexpressing mice [32]. Collectively, CHEP-induced upregulation of SIRT3 may participate in PGC-1α protein expression, mitochondrial biogenesis, and fiber-type switching to oxidative fibers.

The transcriptional activity of PGC-1α is enhanced by AMPK, which directly activates PGC-1α in skeletal muscle by phosphorylating threonine-177 and serine-538 residues [25]. Furthermore, AMPK increases the activity of PGC-1α through deacetylation by SIRT1 [33]. SIRT3 also enhances the expression of PGC-1α through AMPK phosphorylation [23,24]. In this study, CHEP increased the expression of SIRT3 and PGC-1α, as well as the phosphorylation of AMPK, suggesting that CHEP stimulated SIRT3, AMPK, and PGC-1α signaling pathways.

Reportedly, several natural compounds increase the expression of SIRT3 or the phosphorylation of AMPK to increase PGC-1α protein expression. For example, honokiol, a natural compound found in the bark of magnolia trees, upregulates PGC-1α protein expression by enhancing the protein expression level of SIRT3 and AMPK phosphorylation in Alzheimer’s disease-like cells [24]. Honokiol directly activates SIRT3 and elevates PGC-1α gene expression in cardiomyocytes [30]. Cyanidin-3-glucoside (Cy3G), a polyphenol compound present in fruits and vegetables, increases PGC-1α protein expression in skeletal muscle [34]. AMPK phosphorylation through Ca^2+^/calmodulin-dependent protein kinase kinase activation is reportedly involved in Cy3G effects [34]. Similar to honokiol and Cy3G, CHEP probably upregulates PGC-1α protein expression levels through SIRT3 expression or AMPK phosphorylation.

However, as no experiments based on PGC-1α knock-down or knock-out were performed, this study could not clarify whether CHEP directly acts on PGC-1α, SIRT3, or AMPK. In the future, it will be necessary to investigate whether CHEP-induced changes in mitochondrial content and muscle fiber type would be affected by the lack of PGC-1α, SIRT3, or AMPK.

## 5. Conclusions

CHEP increased mitochondrial content and oxidative muscle fibers by upregulating PGC-1α in skeletal muscles. The increased SIRT3 expression and AMPK phosphorylation may be involved in the effects of CHEP on skeletal muscle. Nutraceuticals such as CHEP, which target skeletal muscle PGC-1α and regulate metabolic pathways, may contribute to the prevention of metabolic diseases as functional foods or supplements.

## Data Availability

The data underlying this article will be shared on reasonable request to the corresponding author.

## Supplementary Materials

The following are available online at https://www.mdpi.com/2072-6643/13/2/497/s1, Figure S1: Total protein amount on the membrane, obtained after Ponceau S staining. Table S1: Body weights, tissue weights and food intake of C57BL/6J mice fed an HC diet (control) or an HC diet containing 0.25% (*w*/*w*) CHEP for 5 weeks.
