# Supplementary material for: Citrus hassaku Extract Powder Increases Mitochondrial Content and Oxidative Muscle Fibers by Upregulation of PGC-1α in Skeletal Muscle
Source: Nutrients. 2021 Feb 3;13(2):497. doi: 10.3390/nu13020497 (PMC7913372; doi:10.3390/nu13020497)
Supplement: Supplementary file 1 [file nutrients-13-00497-s001.pdf]

Supplementary Materials

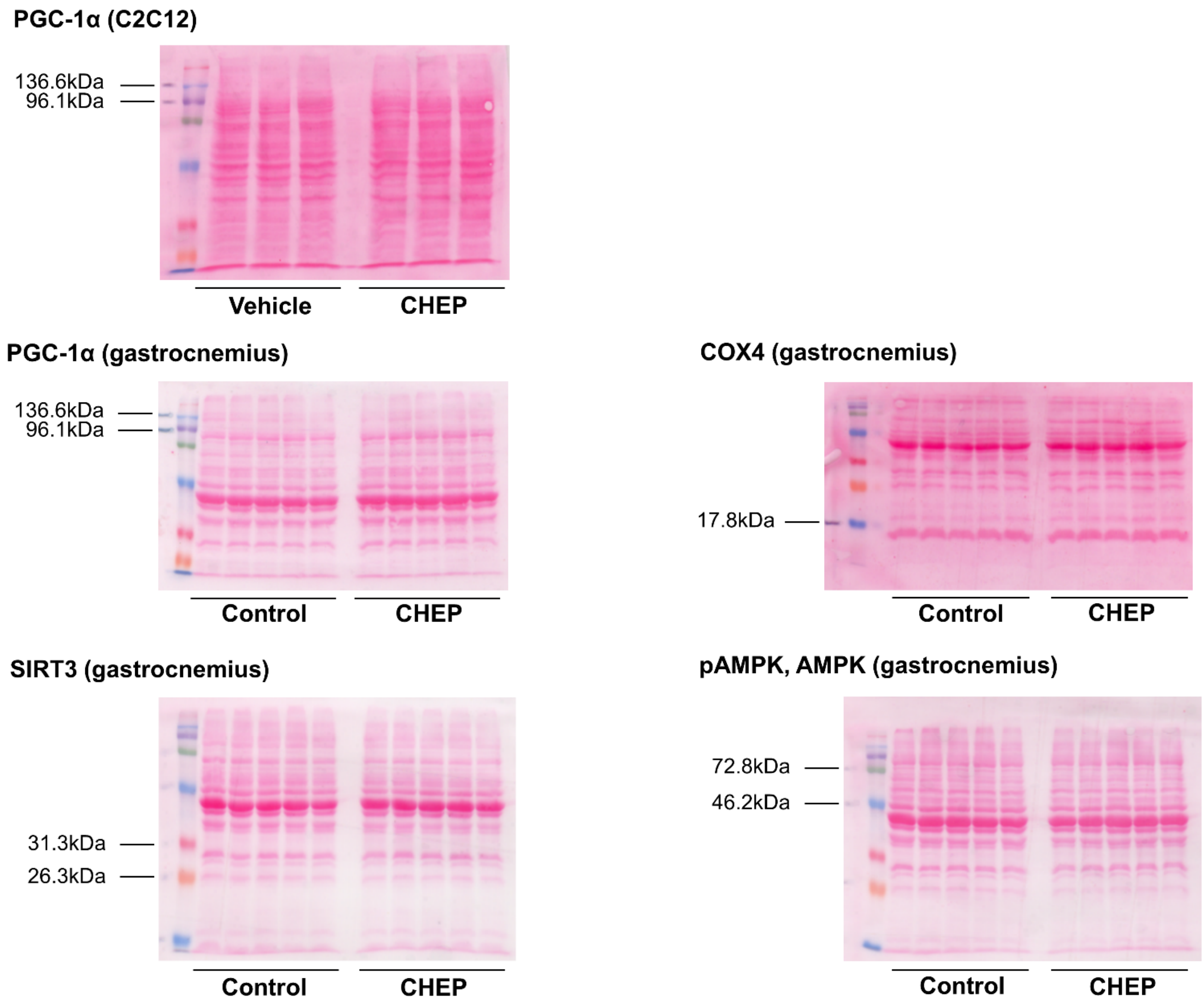

Figure S1. Total protein amount on the membrane, obtained after Ponceau S staining. C2C12 myotubes were incubated with vehicle (DMSO) or 37 μg/mL CHEP for 24 h. Seven-week-old male C57BL/6J mice were treated with an HC diet (control) or an HC diet containing 0.25% (w/w) CHEP for 5 weeks. CHEP, *Citrus hassaku* extract powder; HC, high carbohydrate; PGC-1α, peroxisome proliferator-activated receptor-γ coactivator-1α; COX4, cytochrome c oxidase subunit IV; SIRT3, sirtuin 3; pAMPK, phospho-AMP-activated protein kinase.

Table S1. Body weights, tissue weights, and food intake of C57BL/6J mice fed an HC diet (control) or an HC diet containing 0.25% (w/w) CHEP for 5 weeks.

	Control	CHEP
Body weight (g)	26.6 ± 0.85	24.8 ± 0.47
Tissue weight (g)		
tibialis anterior	0.044 ± 0.0011	0.041 ± 0.0012
extensor digitorum longus	0.010 ± 0.0007	0.009 ± 0.0009
soleus	0.008 ± 0.0003	0.007 ± 0.0002
gastrocnemius	0.134 ± 0.0035	0.129 ± 0.0018
quadriceps	0.138 ± 0.0031	0.140 ± 0.0064
Food intake (g/mouse/day)	3.71	3.38

The quantity of food intake is shown as g/mouse/day, i.e., the total values for each cage divided by the number of mice in the same cage. The other values are expressed as mean ± SEM (n = 5 at 12 weeks of age). No statistically significant difference is observed for all values between the groups. HC, high carbohydrate; CHEP, *Citrus hassaku* extract powder; SEM, standard error of the mean.
